# Evaluation of Dioxin/Furan and Elements in Poultry from Zarqa Governorate, Jordan

**DOI:** 10.1155/2023/8458678

**Published:** 2023-10-13

**Authors:** Mohanad Masad, Samer Alawaideh, Basheer Nusairat, Ali Alnawaiseh, Ahmad Al Shra'ah

**Affiliations:** ^1^Chemistry Department, Al al-Bayt University, Al-Mafraq 25113, Jordan; ^2^Chemistry Department, Isra University, Amman, Jordan; ^3^Jordan University for Science and Technology, Ramtha, Jordan; ^4^Chemistry Department, Tafila Technical University, Tafila, Jordan

## Abstract

This study is the first to determine the concentration for 17 congeners of polychlorinated dibenzo-p-dioxins and polychlorinated dibenzofurans (PCDD/Fs) and element contamination in poultry that is close to petroleum refinery at Al-Hashemiya Municipality, Zarqa Governorate, Jordan. Ten different samples (chicken) were collected to cover ten different locations of poultry farms in Al-Hashemiya Municipality. These locations are considered polluted areas as a result of exhaust gases produced from the refinery. The 17 PCDD/Fs congeners and elements of Pb, Cd, As, Zn, Cu, Se, Hg, Cr, and Ni were determined for three parts of each sample (liver, muscle, and gizzard). All samples were analyzed for PCDD/Fs after a Soxhlet extraction procedure and cleanup by column chromatography; then, all compounds were identified and determined using GC-MS techniques. The elements were analyzed after digestion and measured using an inductively coupled plasma optical emission spectrometer (ICP-OES) and validated with the Lab Mix24 RM NCS ZC73016 reference material. The highest total sum concentration of PCDD/Fs was found in liver samples to be 214.07 ng/kg (dry weight), while the highest sum of toxicity equivalent to PCDD/Fs of 22.54 ng TEQ/kg was found in gizzard samples. For element concentrations, the highest total sum of 16.89 mg/kg (dry weight) was found in liver samples. The concentration level of the elements of Se, Hg, Cr, and Ni for all parts of the chicken was within an acceptable range according to Jordanian standards and therefore the measured level of heavy and trace elements in the poultry samples (chicken) does not pose a danger to public health. The chickens found in poultry farms near the refinery are more likely to contain a higher concentration of PCDD/Fs congeners due to exhaust gas exposure.

## 1. Introduction

Various industrial activities contribute to widespread contamination of the environment by different classes of persistent organic pollutants (POPs), such as polychlorinated dibenzo-p-dioxins and furans (PCDD/Fs). Among these, the oil industry has shown to have a considerable environmental impact on different ecosystems [1]. PCDD/Fs compounds can bioaccumulate and biomagnify in the food chain and the human body. Some of these compounds, such as PCBs, may persist in the environment for periods of years and may bioconcentrate by factors up to 70,000 times [2]. PCDD/Fs belong to the most toxic environmental organic pollutants and can cause serious public health problems [3, 4]. The measurements of industry-posed PCDD/Fs pollutants were considered in many studies, and the negative health effects have been presented [3, 5]. The qualitative and quantitative evaluation of PCDD/Fs in urban air and soils has been studied, and results demonstrated the need of decreasing both the concentration and toxicity of PCDD/Fs [6].

In addition, the concentration levels of elements, namely, lead (Pb), cadmium (Cd), arsenic (As), zinc (Zn), copper (Cu), selenium (Se), mercury (Hg), chromium (Cr), and nickel (Ni), should always be monitored to assess the risk to human health and dietary intake. These elements can be taken up from soil to plants to animals, and finally to humans. Poultry is a major food source in the Middle East, especially in Jordan, and considered as a significant source of human exposure to elements. Food rarely causes acute intoxications, but As, Pb, and Cd accumulate in the body with possible subclinical adverse effects [7]. According to the World Health Organization (WHO), low levels of some elements such as Hg, Cd, Pb, and As can cause diseases in humans (WHO 1977, 1992, and 2001). Trace elements, such as manganese (Mn), cobalt (Co), selenium (Se), molybdenum (Mo), and antimony (Sb), may also be present in high concentrations in food and in excessive dietary intake. The dietary intake of metals in the diet of major food products was determined by the National Library of Medicine [8], and these elements can also have adverse effects on health [9, 10].

Many studies were conducted in many countries to determine the levels of heavy metals and trace elements in poultry and livestock meat, e.g., Holland, Germany, USA, Canada, Spain, Finland [11], Sweden [11, 12], Kazakhstan [13], Egypt [14], Italy [15], Nigeria [16], Chile [17], Turkey [18], Brazil [19], India [20], Taiwan [9], and China [21]. Al-Hashemiya Municipality, Zarqa Governorate, Jordan, is close to the refinery and is considered a highly polluted region. The exhaust gases from the refinery contain toxic materials, which settle in plants, soil, water, and livestock that affect humans. The aim of this study is to evaluate the concentrations of Pb, Cd, As, Zn, Cu, Se, Hg, Cr, and Ni the congeners of PCDD/Fs in poultry (chicken) that was consumed in Al-Hashemiya Municipality, Zarqa Governorate, Jordan.

## 2. Materials and Methods

### 2.1. Chemical Reagents and Solvents

All chemicals used in the digestion process are extra pure: hydrochloric acid (36.5–38.0%); hydrofluoric acid and nitric acid (69.0–71.0%) (Merck Sigma-Aldrich, USA); optima grade hydrogen peroxide (H_2_O_2_) assay (30–32%) (Fisher Scientific, UK); DIW-ASTM Type I (deionized water, ultrapure water, Type I water is defined by the American Society for Testing and Materials (ASTM)).

The chemical products used for extraction and cleaning were purchased as follows: Florisil® 0.15–0.25 mm (60–100 mesh) from Merck, Germany; silica gel 60, 0.063–0.200 mm (70–230 mesh) from Merck, Germany; and extra pure anhydrous sodium sulfate (Fluka, Switzerland). Solvents used are as follows: dichloromethane of 97% purity, n-hexane of 97% purity, and toluene of 98% purity were purchased from Tedia, USA; petroleum ether (40–60°C) of 95% purity was purchased from Carlo Erba, Italy; ethanol absolute of 99% purity, n-nonane of 98.7% purity, and isooctane of 98.7% purity were purchased from Riedel de-Haën, Hannover, Germany; acetone of 99% purity was purchased from GCC, UK.

### 2.2. Standards and Internal Standards

Standard solutions: for the metal calibration curve, the multielement standard solutions (100 *μ*g/mL) (PerkinElmer, Waltham, MA, USA) were used, while for validation, the certified reference material NCS ZC73016 (Lab Mix24, Germany) was used. While AOAC 2015.01 method was used for element analysis. Although the 1613 EPA method stock solution of PCDD/Fs and the internal standard for PCDD/Fs (1,2,3,4-tetrachloro[^13^C_12_] dibenzo-p-dioxin), 200 ng/mL, were purchased from Willington Laboratory, Canada.

### 2.3. Instrumentation

Inductively coupled plasma optical emission spectrometer (ICP-OES) (PerkinElmer) was used for the element determination and interfaced with the computer for calculations, integration, and calibration. A microwave digestion system (Bioevopeak, USA) was used for sample digestion. The instrument gas chromatograph-mass spectrometer (GC/MS), type Shimadzu QP 2010, was used for the determination of OCPs, PCBs, and PCDD/Fs. This instrument was equipped with a split-splitless injector, auto sampler, and quadruple mass selective detector. GC/MS was interfaced with computer for calculations, integration, and calibration.

Analytical balance, hot plate with stirrer, drying oven, water bath, and sonicator were used. All glassware was washed with detergent and water, soaked with 2% (v/v) nitric acid overnight, and rinsed with water and dried.

### 2.4. Sampling and Study Area

**Figure 1:**
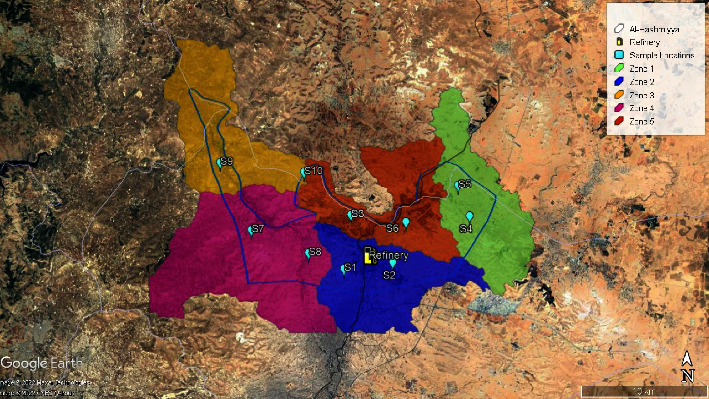
Sample locations and environment.

Ten samples were collected from ten poultry farms located in different places in the Al-Hashemiya Municipality, which is considered a polluted area due to the exhausted gases produced from the refinery. Elements and PCDD/Fs were determined for the liver, muscles, and gizzard of each chicken. The locations and surroundings of the samples are shown in Figure 1.

### 2.5. Sample Preparation

For elements: 0.25 g wet sample was introduced into a microwave digestion vessel, then 4 mL of concentrated HNO_3_ and 1 mL of 30% hydrogen peroxide were added to each digestion vessel. The samples were digested at a minimum temperature of 190°C for a minimum time of 10 min gradually until the vapor and acid fluid inside the vessel became clear. The vessel was cooled to room temperature and the content was poured into an acid-free 50 mL HDPE centrifuge tube and diluted with deionized water, acidified with 1% (v/v) HNO_3,_ to a final volume of 20 mL. Pb, Cd, As, Zn, Cu, Se, Hg, Cr, and Ni were analyzed using ICP-OES using an internal standard solution of 40 *μ*g/mL rhodium (Rh). The dynamic range for the calibration curve was obtained with concentrations between 0.05 and 20 *µ*g/mL. Recoveries were tested on spiked samples at 100, 200, and 500 *µ*g/kg.

For PCDD/Fs [2]: 1.0 g of each homogenized sample was weighed in a Soxhlet thimble, spiked with 200 *μ*L IS (100 ppb), and then extracted in a Soxhlet apparatus with 200 mL toluene for 16 h. The volume of the extracts was reduced to 3 mL and evaporated using the rotary evaporator at 40°C and 240 mbar. The residues were cleaned up on three columns as follows:

Macro alumina column: A glass column (30 × 0.8 cm) was filled with 25 g of Alumina B, super I (basic), and 10 g of sodium sulfate. The residues from the extraction step were added and washed with 80 mL n-hexane, 100 mL n-hexane/dichloromethane (98 : 2%), and 180 mL n-hexane/dichloromethane (50 : 50%) successively. The last fraction was evaporated using a rotary evaporator (40°C, 280 mbar) at a few milliliters.

Mixed silica gel column: A glass column (30 × 0.8 cm) was filled from the bottom upward with 10 g of anhydrous sodium sulfate, 2 g of neutral silica gel, 10 g of acidic silica gel, 2 g of neutral silica gel, 5 g of basic silica gel, and 2 g of neutral silica gel. The residues from the last step were added, and the column was washed with 250 mL n-hexane. The eluates were evaporated using a rotary evaporator at 40°C and 280 mbar to 1-2 mL.

Mini alumina column: A small glass column (10 × 0.8 cm) was filled with 4 g of basic alumina and 2.5 g of sodium sulfate and then the residues from the last step were added. The column was washed with 20 mL of n-hexane/dichloromethane (98 : 2) and then with 35 mL n-hexane/dichloromethane (1 : 1). The last fraction was evaporated using the rotary evaporator to 1-2 mL at 40°C and 280 mbar. The last few milliliters were evaporated using a gentle stream of nitrogen, and the residues were dissolved in 200 *µ*L n-nonane.

## 3. Method Validation

### 3.1. Detection Limits and Limits of Quantitation

The method limit of detection (MLOD) was obtained for each element taking 0.25 g of blank sample and spiked with multistandard solution and measured 10 times (*n* = 10). While for PCDD/Fs, by taking 1.0 g of dry weight of blank chicken samples and mixed with 5.0 g of anhydrous sodium sulfate and spiked with the standard solution. From the average value, the method limit of detection (MLOD) and method limit of quantitation (MLOQ) were calculated when S/N ratio is equal to 3 and 10, respectively. Tables 1 and 2 show the MLOD and MLOQ for elements and PCDD/Fs, respectively.

### 3.2. Instrument Precision

The precision of the ICP-OES instrument was measured through injections using 0.1 *μ*g/mL of multielement standard solution three times and, for GS-MS instrument, was measured through injections three times using PCDD/Fs: (10 ng/mL) for tet-PCDD/Fs, (50 ng/mL) pent, hex, hep-PCDD/Fs, and (100 ng/mL) oct-PCDD/Fs. Coefficients of variation (CV) were found to be within the acceptable limits for trace analysis (CV < 15%) [22].

### 3.3. Recovery

For elements, blank spiked poultry (chicken) samples (liver, mussel, and gizzard) with a concentration of 100, 200, and 500 *µ*g/kg concentration were analyzed, and for PCDD/Fs, a standard solution mixture: (10 ng/mL) for tet-PCDD/Fs, (50 ng/mL) pent, hex, hep-PCDD/Fs, and (100 ng/mL) oct-PCDD/Fs. Recovery was carried out in triplicates at different times, and the average recoveries were calculated. A comparison was made between measured and certified values of the elements against RM Lab Mix24 NCS ZC73016 according to the method mentioned above, as shown in Table 3. For PCDDs/Fs, the average recovery found was tet-PCDD (104%), tet-PCDF (107%), pen-PCDF (101%), pen-PCDD (83%), hex-PCDF (93%), hex-PCDD (85%), hep-PCDD (85%), hep-PCDF (109%), and oct-PCDD/Fs (103%).

## 4. Results and Discussion

### 4.1. Elements

**Figure 2:**
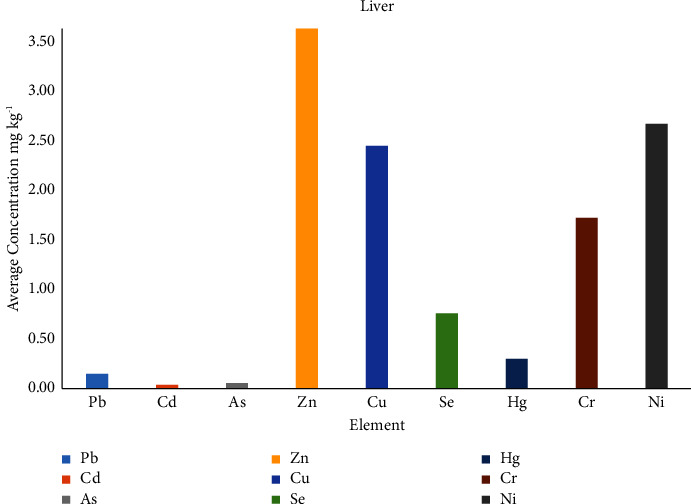
Average elements' concentration (mg/kg) in liver samples.

Zn was found in high concentrations for all samples and the highest sum was 53.37 mg/kg in muscle samples. In liver samples, the highest sum of elements was found to be 16.89 mg/kg for sample S6, and the ranges of element concentration (in mg/kg) are as follows: Pb (0.05–0.24), Cd (0.03-0.04), As (0.04–0.06), Zn (1.42–5.85), Cu (1.46–4.00), Se (0.37–0.95), Hg (0.02–0.53), Cr (0.42–3.12), and Ni (0.94–4.42).  Table 4 shows the average concentration and the sum of all elements for each liver sample, while the distribution of the elements is shown in Figure 2.

**Figure 3:**
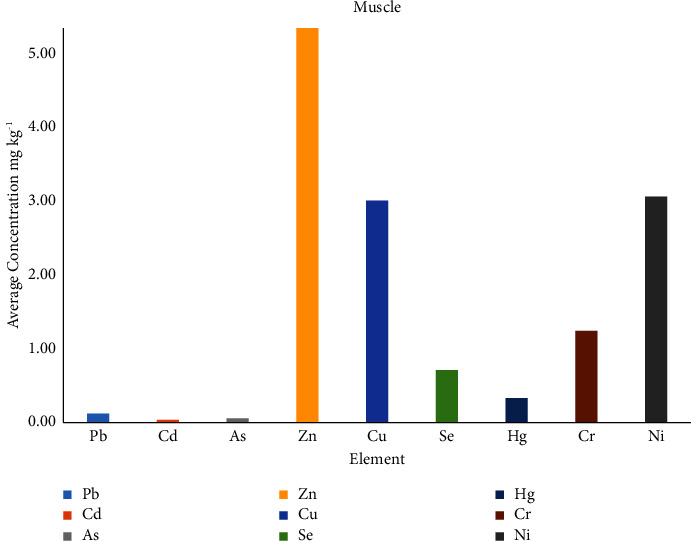
Average elements' concentration (mg/kg) in muscle samples.

In muscle samples, the highest sum of elements was found for sample S3 16.85 mg/kg, and the ranges of element concentration (in mg/kg) found in Pb (0.05–0.23), Cd (0.03–0.05), As (0.04–0.07), Zn (2.44–6.69), Cu (1.74–4.04), Se (0.40–0.94), Hg (0.10–0.54), Cr (0.39–2.31), and Ni (0.70–5.40). The highest sum of all elements found for Zn in muscle samples 53.37 mg/kg.  Table 5 shows the average concentration and the sum of the nine elements for each muscle sample, while the distribution of the elements is shown in Figure 3.

**Figure 4:**
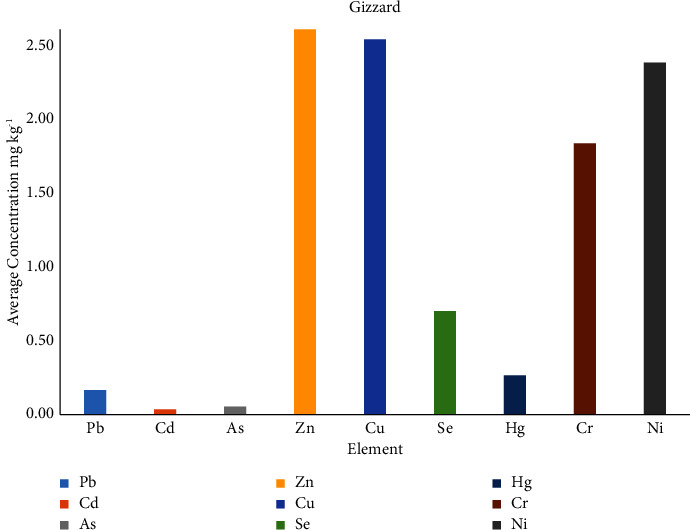
Average elements' concentration (mg/kg) in gizzard samples.

In the gizzard samples (Table 6; Figure 4), the highest sum of the nine elements was found for sample 1, and the ranges of elements' concentration (in mg/kg) found are Pb (0.07–0.24), Cd (0.03-0.04), As (0.04–0.07), Zn (1.29–6.89), Cu (1.29–4.08), Se (0.31–0.87), Hg (0.05–0.51), Cr (0.56–2.43), and Ni (0.63–4.28).

The calculated MLOD and MLOQ for Pb, Cd, As, Zn, Cu, Se, Hg, Cr, and Ni are between 0.03 and 0.1 (mg/kg) and between 0.11 and 0.35 (mg/kg), respectively, as shown in Table 1, while the precision was found to be between 5.07% and 9.75%, which falls within the acceptable values for trace analysis (CV < 15%) [22].

All recoveries for all elements analyzed were found to fall within the reported acceptable range of 80–120% [22]. In liver samples, recovery values were as follows: 89.8–112.5% for Pb, 72.4–82.6% for Cd, 89.0–98.0% for As, 77.3–83.89% for Zn, 97.1–105.3% for Cu, 63.6–99.8% for Se, and 102.5–113.5% for Hg, while in the muscle samples the values were as follows: 87.6–93.5% for Pb, 93.5–105.5% for Cd, 113.5–119.9% for As, 112.7–118.1% for Zn, 96.8–106.2% for Cu, 76.8–91.8% for Se, and 89.8–11.1% for Hg. In the gizzard samples, the recovery values were 81.7–95.5% for Pb, 75.7–88.3% for Cd, 93.2–116.1% for As, 99.3%–117.3% for Zn, 99.8–105.8% for Cu, 66.9–96.5% for Se, and 92.8–106.8% for Hg. All concentration measured values in all samples were compared to the Lab Mix24 NCS ZC73016 reference material and were found to be within <10%, as shown in Table 3, indicating the validity of the analysis method and the results obtained.

Table 7 shows a comparison between the level of elements found in chicken meat from Jordan and other countries [9, 23–25].

### 4.2. Polychlorinated Dibenzo-p-Dioxins (PCDDs)/Polychlorinated Dibenzo Furans (PCDFs)

1,2,3,4,7,8-Hexachlorodibenzofuran was found in high concentrations for all samples with a value of 522.61 ng/kg in liver samples. In liver samples, the highest sum of PCDD/F congeners was found at 214.07 ng/kg for sample S6, and the ranges of PCDD/F congeners (in ng/kg) are as follows: tet-PCDD (0.31–0.72), tet-PCDF (7.47–12.99), pen-PCDF (4.58–26.39), pen-PCDD (1.0–2.30), hex-PCDF (1.46–60.61), hex-PCDD (0.9–7.29), hep-PCDD (5.19–10.61), hep-PCDF (2.23–25.47), and oct-PCDD/F (0.36–2.20).

**Figure 5:**
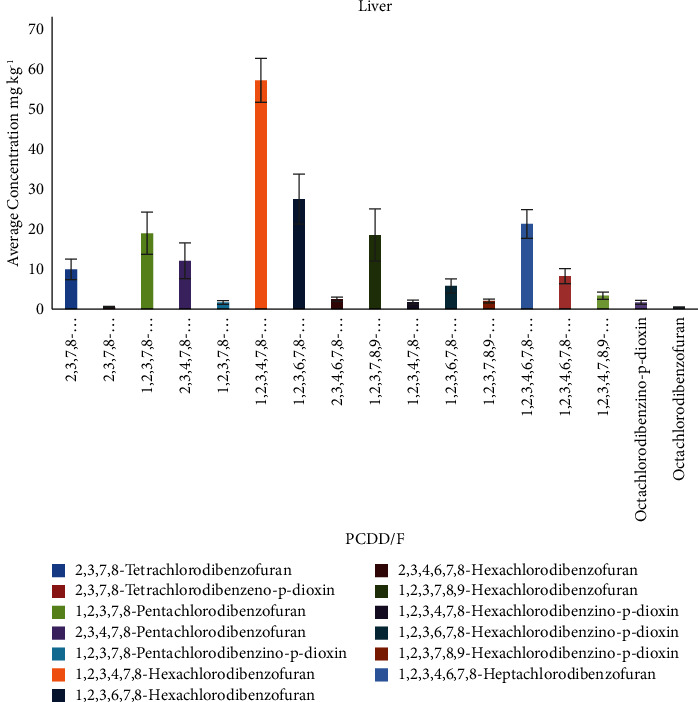
Average concentration of PCDD/Fs congeners (ng/kg) in liver samples.

Table 8 shows the average concentration of all congeners for each liver sample, while the distribution is shown in Figure 5.

**Figure 6:**
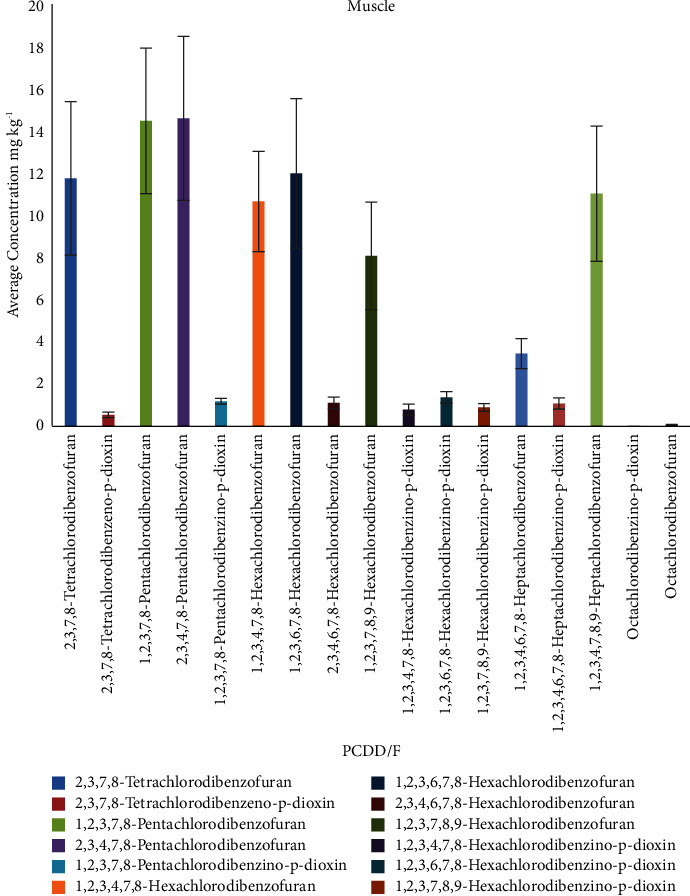
Average concentration of PCDD/Fs congeners (ng/kg) in muscle samples.

In muscle samples, the highest sum of PCDD/F congeners was found for sample S2 104.72 ng/kg, and the highest congener concentration was found for 1,2,3,7,8-pentachlorodibenzofuran with value 130.56 ng/kg. The congener concentration ranges (in ng/kg) found in tet-PCDD (0.31–0.75), tet-PCDF (7.7–17.75), pen-PCDF (7.20–18.32), pen-PCDD (0.93–1.37), hex-PCDF (0.81–15.28), hex-PCDD (0.62–1.74), hep-PCDD (0.62–1.37), hep-PCDF (6.96–16.02), and oct-PCDD/F (0.06–0.12).  Table 9 shows the average concentration of the PCDD/F congeners for each muscle sample, while the distribution of the congeners is shown in Figure 6.

**Figure 7:**
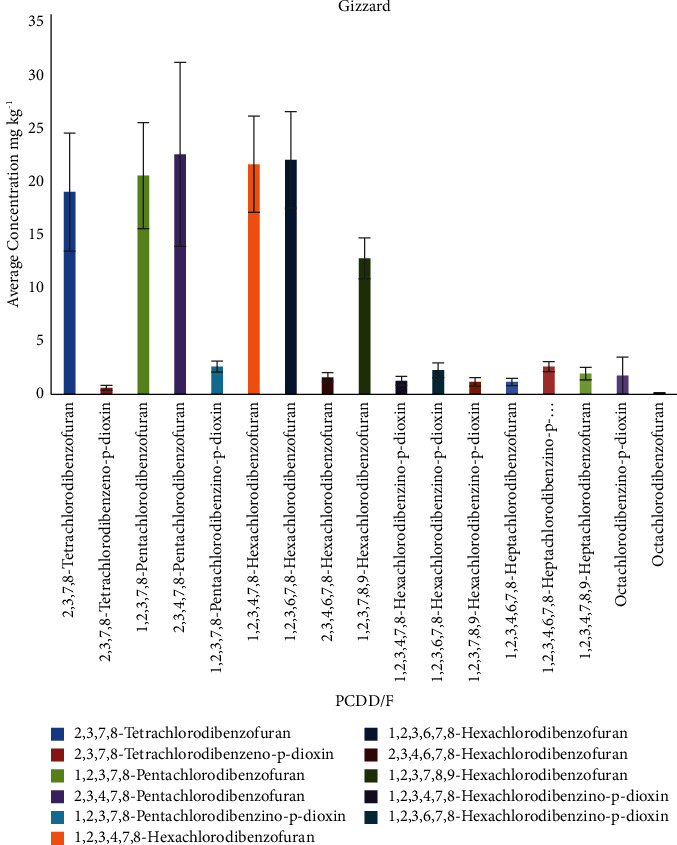
Average concentration of PCDD/Fs congeners (ng/kg) in gizzard samples.

In the gizzard samples (Table 10; Figure 7), the highest sum of PCDD/F congeners was found for sample 4 157.02 ng/kg, and the highest congener concentration was found for 2,3,4,7,8-pentachlorodibenzofuran 204.93 ng/kg. The ranges of PCDD/F congeners average concentration (in ng/kg) found areas follows: tet-PCDD (2.30–3.35), tet-PCDF (10.68–25.84), pen-PCDF (15.16–33.20), pen-PCDD (1.74–3.35), hex-PCDF (0.99–29.07), hex-PCDD (0.62–3.42), hep-PCDD (1.80–3.04), hep-PCDF (1.12–2.61), and oct-PCDD/F (0.06–1.74).

The calculated MLOD and MLOQ for PCDD/F congeners are between 0.02 and 0.03 (ng/kg) and between 0.07 and 0.10 (ng/kg), respectively, as shown in Table 2, while precision was found to be between 5.07% and 9.75%, which falls within the acceptable values for the trace analysis (CV < 15%) [22].

The recoveries for all analyzed PCDD/Fs congeners fall within the reported acceptable range of 80–120% [22]. Average recovery concentrations for congeners in ng/kg were found for tet-PCDD (104%), tet-PCDF (107%), pen-PCDF (101%), pen-PCDD (83%), hex-PCDF (93%), hex-PCDD (85%), hep-PCDD (85%), hep-PCDF (109%), and oct-PCDD/F (103%).

The TEQ (ng/kg) for PCDD/Fs congeners was found as follows: in liver samples, the highest sum was found for sample S6 20.75 ng TEQ/kg. For muscle samples, the highest sum was 12.58 ng TEQ/kg for sample S7. While, in gizzard, the highest sum of TEQ values for PCDD/Fs congeners was found to be 22.54 ng TEQ/kg for sample S3 and was considered the highest between all samples. Tables 11–13 show the average TEQ concentrations (ng TEQ/kg) for liver, muscle, and gizzard samples, respectively.

## 5. Conclusions

The average concentration levels of Se, Hg, Cr, and Ni elements in samples S3, S5, and S6 show a higher value in all parts of chicken, while the average concentrations for PCDD/Fs congers for samples S6, S2, and S4 found the highest sum concentrations due to the proximity of the farms to the refinery and lies to the northeast of wind direction. The level of pollution obtained in chickens in poultry farm samples, for metals, according to Jordanian standards of Codex General Standard for Contaminants and Toxins in Food and Feed (Codex Stan 1993–1995) did not pose a danger to public health in the Al-Hashemiya Municipality in Jordan with a range of 0.01–1.0 mg/kg for poultry meat. The measured PCDD/Fs pollution level, expressed as the sum of all 17 congeners, against the WHO-TEQ approved limit (< 1.0 *μ*g/kg) shows that chickens close to the refinery, where more exhaust gas exposure, contain a the higher concentration of congeners. The preliminary results of this study will be available to environmentalists officials in Jordan to reduce the emission of exhaust gases from the petroleum refinery. The findings of this work suggest that more studies should be conducted within the study area to mitigate the health effect on people who live around the refinery region.

## Figures and Tables

**Table 1:** Method limit of detection (MLOD) and method limit of quantitation (MLOQ) for elements^a^.

Metal	Wavelength (nm)	MLOD (*µ*g/mL)	MLOQ (*µ*g/mL)
Pb	220.353	0.1	0.35
Cd	214.44	0.05	0.18
As	188.979	0.03	0.11
Zn	206.2	0.1	0.35
Cu	327.393	0.08	0.27
Se	196.026	0.03	0.11
Hg	253.652	0.05	0.18
Cr	267.716	0.05	0.18
Ni	231.604	0.03	0.11

^a^Concentric nebulizer with axial view optical system.

**Table 2:** Method limit of detection (MLOD) and method limit of quantitation (MLOQ) for PCDD/Fs.

Peak no.	PCDD/F congeners	Retention time (*t*_*R*_/min)	MLOD (ng/kg dw)	MLOQ (ng/kg dw)
1	2,3,7,8-Tetrachlorodibenzofuran	18.05	0.02	0.07
2	2,3,7,8-Tetrachlorodibenzeno-p-dioxin	18.35	0.03	0.10
3	1,2,3,7,8-Pentachlorodibenzofuran	19.97	0.03	0.10
4	2,3,4,7,8-Pentachlorodibenzofuran	20.40	0.03	0.10
5	1,2,3,7,8-Pentachlorodibenzo-p-dioxin	20.60	0.02	0.07
6	1,2,3,4,7,8-Hexachlorodibenzofuran	22.51	0.02	0.07
7	1,2,3,6,7,8-Hexachlorodibenzofuran	22.63	0.02	0.07
8	2,3,4,6,7,8-Hexachlorodibenzofuran	23.11	0.02	0.07
9	1,2,3,7,8,9-Hexachlorodibenzofuran	23.25	0.02	0.07
10	1,2,3,4,7,8-Hexachlorodibenzo-p-dioxin	23.34	0.02	0.07
11	1,2,3,6,7,8-Hexachlorodibenzo-p-dioxin	23.67	0.02	0.07
12	1,2,3,7,8,9-Hexachlorodibenzo-p-dioxin	23.87	0.02	0.07
13	1,2,3,4,6,7,8-Heptachlorodibenzofuran	25.85	0.02	0.07
14	1,2,3,4,6,7,8-Heptachlorodibenzo-p-dioxin	27.29	0.02	0.07
15	1,2,3,4,7,8,9-Heptachlorodibenzofuran	27.87	0.02	0.07
16	Octachlorodibenzo-p-dioxin	32.84	0.02	0.07
17	Octachlorodibenzofuran	32.99	0.02	0.07

**Table 3:** Determination of elements in chicken with comparison with reference material (Lab Mix24 NCS ZC73016).

Metal	Found (mg/Kg)	Certified values (mg/kg)
Pb	0.11 ± 0.013	0.11 ± 0.02
Cd	0.005 ± 0.01	0.005 ± 3.09
As	0.109 ± 0.03	0.109 ± 0.013
Zn	26 ± 0.32	26 ± 1.0
Cu	1.46 ± 0.09	1.46 ± 0.12
Se	0.109 ± 0.02	0.109 ± 0.013
Hg	3.6 ± 1.31	3.6 ± 1.50
Cr	0.59 ± 0.19	0.59 ± 0.11
Ni	0.15 ± 0.05	0.15 ± 0.03

**Table 4:** The average concentration and the sum of elements in liver samples (mg/kg).

	Pb	Cd	As	Zn	Cu	Se	Hg	Cr	Ni	∑Element
S1	0.20 ± 0.12	0.04 ± 0.01	0.06 ± 0.04	3.12 ± 1.18	3.86 ± 0.57	0.87 ± 0.12	0.43 ± 0.26	2.17 ± 0.03	2.46 ± 0.89	13.21
S2	0.05 ± 0.03	0.04 ± 0.01	0.04 ± 0.02	3.06 ± 1.62	1.46 ± 0.10	0.66 ± 0.15	0.43 ± 0.24	1.55 ± 0.03	1.90 ± 0.84	9.19
S3	0.23 ± 0.05	0.04 ± 0.01	0.05 ± 0.03	4.58 ± 2.33	4.00 ± 0.17	0.90 ± 0.01	0.12 ± 0.07	0.42 ± 0.20	0.94 ± 0.76	11.28
S4	0.12 ± 0.04	0.04 ± 0.01	0.06 ± 0.03	2.09 ± 0.22	1.67 ± 0.40	0.87 ± 0.45	0.11 ± 0.03	1.06 ± 0.67	3.12 ± 0.28	9.14
S5	0.24 ± 0.12	0.04 ± 0.02	0.05 ± 0.03	5.85 ± 2.91	1.98 ± 0.09	0.80 ± 0.68	0.26 ± 0.03	2.41 ± 0.35	3.05 ± 0.60	14.67
**S6**	0.18 ± 0.09	0.06 ± 0.02	0.08 ± 0.03	6.42 ± 1.14	1.73 ± 0.32	0.77 ± 0.08	0.11 ± 0.01	3.12 ± 0.86	4.42 ± 0.15	**16.89**
S7	0.07 ± 0.04	0.03 ± 0.01	0.06 ± 0.03	3.91 ± 0.16	2.38 ± 0.22	0.88 ± 0.75	0.17 ± 0.03	1.04 ± 0.92	2.91 ± 0.07	11.46
S8	0.18 ± 0.10	0.03 ± 0.01	0.06 ± 0.03	1.95 ± 0.64	1.79 ± 0.71	0.37 ± 0.09	0.53 ± 0.03	2.43 ± 0.76	3.57 ± 0.03	10.91
S9	0.19 ± 0.04	0.04 ± 0.02	0.05 ± 0.03	5.45 ± 0.71	3.72 ± 0.97	0.95 ± 0.08	0.49 ± 0.03	1.03 ± 0.38	1.13 ± 0.04	13.05
S10	0.09 ± 0.01	0.03 ± 0.01	0.05 ± 0.03	4.91 ± 2.05	1.93 ± 0.17	0.71 ± 0.18	0.44 ± 0.03	2.01 ± 0.14	3.22 ± 0.08	13.40
∑Element	1.55 ± 0.23	0.39 ± 0.04	0.57 ± 0.10	**41.34** ± 4.94	24.52 ± 1.47	7.78 ± 1.15	3.09 ± 0.37	17.24 ± 1.71	26.72 ± 1.60	123.20

The number given in bold is to show the highest sum between all.

**Table 5:** The average concentration and the sum of elements in muscle samples (mg/kg).

	Pb	Cd	As	Zn	Cu	Se	Hg	Cr	Ni	∑Element
S1	0.08 ± 0.05	0.05 ± 0.03	0.07 ± 0.03	6.40 ± 0.35	2.17 ± 0.48	0.85 ± 0.44	0.19 ± 0.02	0.39 ± 0.03	0.76 ± 0.06	10.96
S2	0.05 ± 0.03	0.03 ± 0.01	0.05 ± 0.03	4.49 ± 0.02	4.04 ± 0.66	0.80 ± 0.38	0.46 ± 0.01	0.83 ± 0.13	5.04 ± 0.27	15.79
**S3**	0.23 ± 0.13	0.03 ± 0.01	0.05 ± 0.03	5.81 ± 0.78	2.97 ± 0.01	0.86 ± 0.71	0.54 ± 0.36	2.16 ± 0.59	4.20 ± 0.12	**16.85**
S4	0.10 ± 0.07	0.04 ± 0.03	0.07 ± 0.02	6.34 ± 0.36	3.50 ± 0.54	0.44 ± 0.03	0.23 ± 0.05	1.01 ± 0.70	0.75 ± 0.03	12.48
S5	0.06 ± 0.03	0.04 ± 0.03	0.06 ± 0.03	2.68 ± 0.62	3.91 ± 0.8	0.68 ± 0.17	0.45 ± 0.09	0.57 ± 0.06	0.70 ± 0.15	9.16
S6	0.15 ± 0.03	0.03 ± 0.01	0.07 ± 0.03	5.77 ± 0.02	3.23 ± 0.21	0.76 ± 0.19	0.10 ± 0.07	1.74 ± 0.09	4.90 ± 0.92	16.75
S7	0.19 ± 0.03	0.03 ± 0.01	0.04 ± 0.01	6.53 ± 0.16	3.29 ± 0.68	0.94 ± 0.67	0.20 ± 0.04	0.94 ± 0.18	3.68 ± 0.94	15.85
S8	0.05 ± 0.03	0.04 ± 0.03	0.05 ± 0.03	6.69 ± 0.79	1.77 ± 0.32	0.91 ± 0.33	0.39 ± 0.07	0.76 ± 0.03	3.51 ± 0.28	14.17
S9	0.20 ± 0.09	0.04 ± 0.02	0.04 ± 0.03	2.44 ± 0.23	3.43 ± 0.14	0.40 ± 0.08	0.31 ± 0.07	2.31 ± 0.27	4.75 ± 0.84	13.92
S10	0.10 ± 0.03	0.04 ± 0.03	0.07 ± 0.04	6.22 ± 0.39	1.74 ± 0.77	0.45 ± 0.01	0.45 ± 0.29	1.71 ± 0.98	2.29 ± 0.11	13.07
∑Element	1.22 ± 0.19	0.36 ± 0.07	0.58 ± 0.09	**53.37** ± 1.45	30.05 ± 1.68	7.09 ± 1.21	3.32 ± 0.49	12.42 ± 1.39	30.58 ± 1.625	138.98

The number given in bold is to show the highest sum between all.

**Table 6:** The average concentration of elements in gizzard samples (mg/kg).

	Pb	Cd	As	Zn	Cu	Se	Hg	Cr	Ni	∑Element
**S1**	0.07 ± 0.03	0.03 ± 0.01	0.05 ± 0.03	6.89 ± 0.08	2.79 ± 0.07	0.55 ± 0.05	0.51 ± 0.03	0.56 ± 0.06	4.15 ± 0.05	**15.61**
S2	0.12 ± 0.03	0.04 ± 0.03	0.06 ± 0.03	2.18 ± 0.02	2.04 ± 0.09	0.85 ± 0.09	0.48 ± 0.03	0.82 ± 0.08	2.02 ± 0.03	8.61
S3	0.21 ± 0.03	0.04 ± 0.03	0.06 ± 0.02	1.41 ± 0.23	3.47 ± 0.07	0.65 ± 0.08	0.05 ± 0.03	2.21 ± 0.07	1.24 ± 0.09	9.35
S4	0.14 ± 0.03	0.04 ± 0.03	0.06 ± 0.03	2.46 ± 0.12	1.37 ± 0.07	0.79 ± 0.07	0.44 ± 0.03	2.43 ± 0.07	2.84 ± 0.06	10.57
S5	0.20 ± 0.03	0.03 ± 0.02	0.04 ± 0.03	1.31 ± 0.11	1.29 ± 0.07	0.56 ± 0.17	0.33 ± 0.07	1.96 ± 0.06	4.28 ± 0.05	10.00
S6	0.16 ± 0.05	0.04 ± 0.03	0.04 ± 0.03	1.29 ± 0.05	4.08 ± 0.12	0.85 ± 0.07	0.21 ± 0.05	2.01 ± 0.05	1.94 ± 0.05	10.62
S7	0.24 ± 0.07	0.03 ± 0.01	0.06 ± 0.03	2.11 ± 0.05	3.26 ± 0.17	0.31 ± 0.07	0.12 ± 0.03	2.32 ± 0.05	2.07 ± 0.05	10.52
S8	0.13 ± 0.03	0.04 ± 0.03	0.07 ± 0.03	3.66 ± 0.05	1.79 ± 0.07	0.86 ± 0.14	0.27 ± 0.09	1.75 ± 0.05	1.96 ± 0.08	10.52
S9	0.19 ± 0.03	0.04 ± 0.02	0.04 ± 0.02	1.35 ± 0.07	3.95 ± 0.27	0.71 ± 0.07	0.17 ± 0.09	2.05 ± 0.04	0.63 ± 0.09	9.12
S10	0.19 ± 0.03	0.04 ± 0.01	0.04 ± 0.03	3.42 ± 0.07	1.37 ± 0.07	0.87 ± 0.13	0.08 ± 0.08	2.26 ± 0.09	2.71 ± 0.09	10.98
∑Element	1.65 ± 0.12	0.35 ± 0.07	0.54 ± 0.09	**26.08** ± 0.32	25.41 ± 0.39	7.00 ± 0.32	2.66 ± 0.19	18.37 ± 0.20	23.84 ± 0.21	105.89

The number given in bold is to show the highest sum between all.

**Table 7:** Comparison between the levels of elements in chicken meat found in Jordan and other countries.

Metal	Taiwan (mg/kg)	Pakistan (mg/kg)	Egypt (mg/kg)	Saudi Arabia (mg/kg)	Jordan (mg/kg)
Pb	0.004	—	0.25	—	0.12
Cd	0.002	0.04	—	—	0.04
As	0.04	—	0.36	—	0.06
Zn	—	—	—	41.40	5.34
Cu	—	0.002	0.15	0.06	3.01
Se	0.35	—	—	—	0.71
Hg	—	—	0.19	—	0.33
Cr	—	0.11	—	9.75	1.24
Ni	—	0.04	—	—	3.06

**Table 8:** Average concentration of PCDD/Fs congeners in liver samples (ng/kg).

	S1	S2	S3	S4	S5	**S6**	S7	S8	S9	S10	∑Congeners
2,3,7,8-Tetrachlorodibenzofuran	11.30 ± 0.42	6.14 ± 0.75	9.46 ± 0.25	12.25 ± 0.75	12.84 ± 0.67	7.47 ± 0.50	10.92 ± 0.50	9.23 ± 0.67	6.45 ± 0.27	12.99 ± 0.25	86.06
2,3,7,8-Tetrachlorodibenzeno-p-dioxin	0.72 ± 0.50	0.72 ± 0.06	0.56 ± 0.07	0.41 ± 0.05	0.72 ± 0.25	0.61 ± 0.25	0.28 ± 0.08	0.46 ± 0.09	0.31 ± 0.03	0.72 ± 0.04	4.78
1,2,3,7,8-Pentachlorodibenzofuran	16.83 ± 0.33	12.30 ± 0.25	16.85 ± 0.42	26.39 ± 0.58	12.23 ± 0.25	24.40 ± 0.50	22.51 ± 0.33	24.25 ± 0.50	14.17 ± 0.42	18.80 ± 0.42	169.92
2,3,4,7,8-Pentachlorodibenzofuran	4.58 ± 0.50	17.14 ± 0.33	16.34 ± 0.50	9.05 ± 0.50	9.95 ± 0.42	13.45 ± 0.42	16.45 ± 0.57	8.82 ± 0.42	8.18 ± 0.38	16.29 ± 0.55	103.96
1,2,3,7,8-Pentachlorodibenzo-p-dioxin	2.30 ± 0.81	1.41 ± 0.42	1.64 ± 0.42	2.05 ± 0.75	1.46 ± 0.25	1.20 ± 0.42	1.43 ± 0.50	2.28 ± 0.08	2.10 ± 0.15	1.00 ± 0.04	15.86
1,2,3,4,7,8-Hexachlorodibenzofuran	56.60 ± 1.83	53.32 ± 0.25	63.02 ± 0.33	59.97 ± 0.51	57.34 ± 0.67	63.25 ± 0.75	56.88 ± 0.42	51.61 ± 0.50	60.61 ± 0.58	45.77 ± 0.42	**522.61**
1,2,3,6,7,8-Hexachlorodibenzofuran	20.28 ± 0.58	20.43 ± 0.75	18.72 ± 0.42	31.99 ± 0.49	26.45 ± 0.42	38.80 ± 0.75	29.57 ± 0.83	27.93 ± 0.58	31.33 ± 0.07	28.18 ± 0.42	245.50
2,3,4,6,7,8-Hexachlorodibenzofuran	2.33 ± 0.67	2.74 ± 0.66	2.51 ± 0.75	1.46 ± 0.37	2.97 ± 0.07	3.12 ± 0.50	3.04 ± 0.65	1.79 ± 0.50	2.38 ± 0.08	2.53 ± 0.33	22.33
1,2,3,7,8,9-Hexachlorodibenzofuran	13.30 ± 0.33	14.96 ± 0.50	21.97 ± 0.33	28.31 ± 0.59	18.80 ± 0.50	18.49 ± 0.50	10.10 ± 0.25	9.92 ± 0.57	20.82 ± 0.35	27.54 ± 0.25	156.68
1,2,3,4,7,8-Hexachlorodibenzo-p-dioxin	1.79 ± 0.58	2.20 ± 0.58	2.56 ± 0.83	0.90 ± 0.25	1.48 ± 0.25	1.89 ± 0.75	2.23 ± 0.33	1.61 ± 0.37	1.61 ± 0.13	1.33 ± 0.05	16.27
1,2,3,6,7,8-Hexachlorodibenzo-p-dioxin	3.09 ± 0.50	6.93 ± 0.67	7.29 ± 0.67	5.60 ± 0.25	7.93 ± 0.25	3.43 ± 0.83	6.34 ± 0.42	6.11 ± 0.25	3.79 ± 0.33	7.47 ± 0.08	50.51
1,2,3,7,8,9-Hexachlorodibenzo-p-dioxin	2.30 ± 0.58	2.15 ± 0.50	1.25 ± 0.42	2.35 ± 0.58	2.38 ± 0.83	1.87 ± 0.35	2.35 ± 0.50	1.53 ± 0.33	2.69 ± 0.15	1.66 ± 0.03	18.87
1,2,3,4,6,7,8-Heptachlorodibenzofuran	16.27 ± 0.50	24.55 ± 0.83	20.31 ± 0.85	15.32 ± 0.59	21.41 ± 0.25	23.43 ± 0.50	22.12 ± 0.50	24.60 ± 0.76	25.47 ± 0.42	18.45 ± 0.13	193.48
1,2,3,4,6,7,8-Heptachlorodibenzino-p-dioxin	9.80 ± 2.75	10.61 ± 0.42	8.29 ± 0.51	10.38 ± 0.67	7.65 ± 0.50	6.24 ± 0.25	7.16 ± 0.25	9.77 ± 0.37	5.19 ± 0.17	7.31 ± 0.25	75.09
1,2,3,4,7,8,9-Heptachlorodibenzofuran	2.30 ± 0.42	4.25 ± 0.25	3.81 ± 0.34	5.09 ± 0.09	2.76 ± 0.67	4.07 ± 0.25	3.12 ± 0.42	2.76 ± 0.25	2.23 ± 0.03	3.20 ± 0.05	30.38
Octachlorodibenzo-p-dioxin	2.20 ± 0.75	2.07 ± 0.33	1.43 ± 0.67	1.89 ± 0.07	0.90 ± 0.08	1.89 ± 0.32	1.66 ± 0.37	1.82 ± 0.25	0.92 ± 0.04	2.30 ± 0.08	14.78
Octachlorodibenzofuran	0.36 ± 0.03	0.64 ± 0.15	0.56 ± 0.07	0.54 ± 0.33	0.46 ± 0.09	0.46 ± 0.15	0.59 ± 0.18	0.54 ± 0.13	0.54 ± 0.05	0.43 ± 0.07	4.68
∑congeners	166.34 ± 3.17	182.56 ± 1.67	196.57 ± 1.88	213.96 ± 1.37	187.70 ± 1.36	**214.07** ± 1.54	196.75 ± 1.30	185.04 ± 1.32	188.77 ± 0.70	195.98 ± 0.52	1731.77

The number given in bold is to show the highest sum between all.

**Table 9:** Average concentration of PCDD/Fs congeners in muscle samples (ng/kg).

	S1	**S2**	S3	S4	S5	S6	S7	S8	S9	S10	∑congeners
2,3,7,8-Tetrachlorodibenzofuran	12.42 ± 1.83	15.16 ± 1.83	8.63 ± 0.55	12.36 ± 0.42	9.81 ± 1.83	16.15 ± 0.25	17.95 ± 0.75	7.70 ± 0.17	8.26 ± 0.11	9.13 ± 0.25	108.45
2,3,7,8-Tetrachlorodibenzeno-p-dioxin	0.50 ± 0.09	0.56 ± 0.06	0.68 ± 0.08	0.31 ± 0.08	0.56 ± 0.06	0.43 ± 0.07	0.43 ± 0.05	0.75 ± 0.09	0.56 ± 0.08	0.62 ± 0.04	4.78
1,2,3,7,8-Pentachlorodibenzofuran	9.69 ± 3.67	19.81 ± 0.50	11.86 ± 0.33	9.38 ± 0.35	18.32 ± 0.45	14.66 ± 0.18	16.71 ± 0.19	13.98 ± 0.11	16.15 ± 0.25	14.35 ± 0.07	**130.56**
2,3,4,7,8-Pentachlorodibenzofuran	15.78 ± 0.25	16.46 ± 0.42	7.20 ± 0.75	17.64 ± 0.35	16.71 ± 0.35	13.85 ± 0.25	17.83 ± 0.55	15.40 ± 0.18	7.95 ± 0.35	17.27 ± 0.13	128.82
1,2,3,7,8-Pentachlorodibenzo-p-dioxin	1.24 ± 0.09	1.30 ± 0.07	1.18 ± 0.05	1.30 ± 0.06	1.37 ± 0.09	1.12 ± 0.06	1.06 ± 0.08	1.12 ± 0.04	0.93 ± 0.07	1.24 ± 0.07	10.62
1,2,3,4,7,8-Hexachlorodibenzofuran	10.99 ± 0.51	9.50 ± 0.09	12.73 ± 0.25	8.63 ± 0.15	15.28 ± 0.08	10.31 ± 0.05	9.44 ± 0.06	9.07 ± 0.08	13.11 ± 0.05	7.58 ± 0.08	99.07
1,2,3,6,7,8-Hexachlorodibenzofuran	10.87 ± 0.33	10.37 ± 0.25	10.87 ± 0.35	17.58 ± 0.25	9.01 ± 0.15	10.19 ± 0.25	9.07 ± 0.06	16.96 ± 0.11	16.46 ± 0.15	8.63 ± 0.05	111.37
2,3,4,6,7,8-Hexachlorodibenzofuran	1.30 ± 0.25	0.93 ± 0.09	0.68 ± 0.25	1.06 ± 0.15	1.37 ± 0.06	1.49 ± 0.33	0.81 ± 0.03	0.93 ± 0.03	1.37 ± 0.05	1.18 ± 0.03	9.94
1,2,3,7,8,9-Hexachlorodibenzofuran	7.08 ± 0.35	8.07 ± 0.25	5.71 ± 0.09	6.02 ± 0.07	9.32 ± 0.05	12.30 ± 0.33	10.75 ± 0.11	6.34 ± 0.06	4.66 ± 0.07	10.62 ± 0.15	70.25
1,2,3,4,7,8-Hexachlorodibenzo-p-dioxin	0.62 ± 0.07	1.18 ± 0.07	0.62 ± 0.11	0.93 ± 0.06	0.50 ± 0.07	0.81 ± 0.33	0.75 ± 0.05	1.24 ± 0.08	0.75 ± 0.05	0.56 ± 0.05	7.39
1,2,3,6,7,8-Hexachlorodibenzo-p-dioxin	1.12 ± 0.05	1.30 ± 0.06	1.37 ± 0.03	1.18 ± 0.11	0.93 ± 0.07	1.74 ± 0.08	1.24 ± 0.05	1.68 ± 0.04	1.74 ± 0.03	1.43 ± 0.03	12.30
1,2,3,7,8,9-Hexachlorodibenzo-p-dioxin	0.99 ± 0.05	0.62 ± 0.08	1.06 ± 0.06	1.06 ± 0.05	1.06 ± 0.03	1.06 ± 0.08	0.93 ± 0.05	0.81 ± 0.05	0.62 ± 0.05	0.75 ± 0.08	8.20
1,2,3,4,6,7,8-Heptachlorodibenzofuran	2.17 ± 0.07	4.29 ± 0.08	4.29 ± 0.09	2.80 ± 0.05	4.04 ± 0.05	3.11 ± 0.06	3.48 ± 0.03	3.73 ± 0.25	2.80 ± 0.11	3.79 ± 0.08	30.68
1,2,3,4,6,7,8-Heptachlorodibenzo-p-dioxin	1.12 ± 0.05	1.43 ± 0.05	0.62 ± 0.06	1.24 ± 0.04	0.93 ± 0.15	1.18 ± 0.13	1.37 ± 0.05	1.12 ± 0.07	0.68 ± 0.05	1.12 ± 0.03	9.69
1,2,3,4,7,8,9-Heptachlorodibenzofuran	6.96 ± 0.06	13.66 ± 0.25	11.99 ± 0.09	7.45 ± 0.04	12.98 ± 0.17	9.81 ± 0.15	9.69 ± 0.13	7.45 ± 0.05	14.29 ± 0.06	16.02 ± 0.15	94.29
Octachlorodibenzo-p-dioxin	ND±	ND±	ND±	ND±	ND±	ND±	ND±	ND±	ND±	ND±	0.00
Octachlorodibenzofuran	0.06 ± 0.03	0.06 ± 0.03	0.06 ± 0.03	0.06 ± 0.04	0.06 ± 0.03	0.06 ± 0.04	0.06 ± 0.03	0.06 ± 0.03	0.06 ± 0.03	0.12 ± 0.03	0.56
∑Congeners	82.92 ± 0.45	**104.72** ± 0.40	79.57 ± 0.33	89.01 ± 0.23	102.24 ± 0.27	98.26 ± 0.62	101.55 ± 0.20	88.32 ± 0.29	90.37 ± 0.18	94.41 ± 0.25	836.96

The number given in bold is to show the highest sum between all.

**Table 10:** Average concentration of PCDD/Fs congeners in gizzard samples (ng/kg).

	S1	S2	S3	**S4**	S5	S6	S7	S8	S9	S10	∑Congeners
2,3,7,8-Tetrachlorodibenzofuran	11.61 ± 0.15	19.63 ± 0.25	25.84 ± 0.15	24.35 ± 0.33	22.34 ± 0.36	20.99 ± 0.25	10.68 ± 0.33	12.42 ± 0.35	22.92 ± 0.42	18.26 ± 0.33	170.79
2,3,7,8-Tetrachlorodibenzeno-p-dioxin	0.68 ± 0.03	0.93 ± 0.07	0.56 ± 0.05	0.37 ± 0.03	0.37 ± 0.08	0.81 ± 0.07	0.62 ± 0.08	0.37 ± 0.04	0.31 ± 0.06	0.93 ± 0.07	5.03
1,2,3,7,8-Pentachlorodibenzofuran	17.27 ± 0.25	14.29 ± 0.25	21.86 ± 0.35	20.99 ± 1.83	20.37 ± 1.83	23.60 ± 0.06	29.57 ± 0.33	16.77 ± 0.25	14.22 ± 0.06	25.40 ± 0.16	178.94
2,3,4,7,8-Pentachlorodibenzofuran	15.78 ± 0.15	14.41 ± 0.25	31.73 ± 0.55	28.01 ± 0.25	33.20 ± 0.55	15.16 ± 0.30	35.40 ± 0.18	15.34 ± 0.83	15.90 ± 0.33	19.32 ± 0.27	**204.93**
1,2,3,7,8-Pentachlorodibenzo-p-dioxin	2.30 ± 0.07	2.30 ± 0.08	3.35 ± 0.03	3.23 ± 0.08	1.99 ± 0.08	2.92 ± 0.07	1.74 ± 0.06	2.48 ± 0.07	2.92 ± 0.06	2.61 ± 0.06	23.23
1,2,3,4,7,8-Hexachlorodibenzofuran	29.07 ± 0.25	19.01 ± 0.40	19.30 ± 0.25	26.15 ± 0.67	18.07 ± 1.83	22.24 ± 0.31	27.52 ± 0.67	18.39 ± 0.67	18.94 ± 0.58	16.27 ± 0.16	198.68
1,2,3,6,7,8-Hexachlorodibenzofuran	16.09 ± 0.15	26.58 ± 0.67	20.60 ± 0.35	29.07 ± 0.33	23.04 ± 2.75	18.51 ± 0.11	16.83 ± 0.33	18.07 ± 0.75	25.34 ± 0.45	24.97 ± 0.18	194.14
2,3,4,6,7,8-Hexachlorodibenzofuran	2.11 ± 0.08	1.43 ± 0.05	2.11 ± 0.08	1.68 ± 0.06	1.68 ± 0.44	1.30 ± 0.08	0.99 ± 0.07	1.24 ± 0.06	2.17 ± 0.07	1.12 ± 0.04	14.72
1,2,3,7,8,9-Hexachlorodibenzofuran	15.47 ± 0.25	12.05 ± 0.17	10.00 ± 0.06	12.36 ± 0.50	13.11 ± 0.25	10.25 ± 0.25	14.60 ± 0.25	15.03 ± 0.66	12.98 ± 0.15	11.18 ± 0.75	115.84
1,2,3,4,7,8-Hexachlorodibenzo-p-dioxin	1.80 ± 0.05	1.49 ± 0.03	1.06 ± 0.07	0.62 ± 0.07	1.49 ± 0.06	1.61 ± 0.05	1.37 ± 0.06	0.75 ± 0.06	0.81 ± 0.03	1.61 ± 0.09	10.99
1,2,3,6,7,8-Hexachlorodibenzo-p-dioxin	2.61 ± 0.03	2.42 ± 0.06	3.11 ± 0.08	1.43 ± 0.05	2.30 ± 0.07	3.42 ± 0.08	1.30 ± 0.06	1.74 ± 0.07	1.74 ± 0.07	2.42 ± 0.11	20.06
1,2,3,7,8,9-Hexachlorodibenzo-p-dioxin	0.87 ± 0.07	1.06 ± 0.03	1.80 ± 0.05	1.74 ± 0.06	0.87 ± 0.05	0.62 ± 0.04	1.37 ± 0.08	0.81 ± 0.08	1.12 ± 0.06	1.37 ± 0.08	10.25
1,2,3,4,6,7,8-Heptachlorodibenzofuran	1.49 ± 0.04	0.81 ± 0.04	1.68 ± 0.04	1.61 ± 0.07	1.18 ± 0.08	1.18 ± 0.03	0.99 ± 0.07	1.06 ± 0.07	0.68 ± 0.07	0.81 ± 0.06	10.68
1,2,3,4,6,7,8-Heptachlorodibenzo-p-dioxin	2.92 ± 0.03	2.98 ± 0.07	2.86 ± 0.07	1.80 ± 0.07	3.04 ± 0.03	2.98 ± 0.03	2.11 ± 0.04	2.61 ± 0.07	2.61 ± 0.08	1.93 ± 0.07	23.91
1,2,3,4,7,8,9-Heptachlorodibenzofuran	1.80 ± 0.05	2.80 ± 0.03	2.17 ± 0.05	2.24 ± 0.04	2.61 ± 0.04	2.30 ± 0.03	1.12 ± 0.07	1.43 ± 0.08	1.24 ± 0.06	1.55 ± 0.06	17.70
Octachlorodibenzo-p-dioxin	1.74 ± 0.07	0.81 ± 0.03	0.81 ± 0.06	1.30 ± 0.04	1.61 ± 0.03	0.87 ± 0.05	1.30 ± 0.08	1.30 ± 0.03	0.99 ± 0.08	0.99 ± 0.09	10.75
Octachlorodibenzofuran	0.06 ± 0.03	0.06 ± 0.03	0.12 ± 0.03	0.06 ± 0.04	0.06 ± 0.04	0.19 ± 0.03	0.19 ± 0.04	0.06 ± 0.03	0.06 ± 0.04	0.06 ± 0.03	0.87
∑Congeners	123.66 ± 0.30	123.04 ± 0.21	148.96 ± 0.19	**157.02** ± 0.53	147.34 ± 0.53	128.94 ± 0.29	147.70 ± 0.32	109.88 ± 0.69	124.97 ± 0.24	130.81 ± 0.78	1211.52

The number given in bold is to show the highest sum between all.

**Table 11:** TEQ ng/kg for PCDD/Fs congeners in liver samples.

	S1	S2	S3	S4	S5	**S6**	S7	S8	S9	S10
2,3,7,8-Tetrachlorodibenzofuran	1.13	0.61	0.95	1.23	1.28	0.75	1.09	0.92	0.64	1.30
2,3,7,8-Tetrachlorodibenzeno-p-dioxin	0.72	0.72	0.56	0.41	0.72	0.61	0.28	0.46	0.31	0.72
1,2,3,7,8-Pentachlorodibenzofuran	0.50	0.37	0.51	0.79	0.37	0.73	0.68	0.73	0.43	0.56
2,3,4,7,8-Pentachlorodibenzofuran	1.37	5.14	4.90	2.72	2.98	4.04	4.93	2.65	2.46	4.89
1,2,3,7,8-Pentachlorodibenzo-p-dioxin	2.30	1.41	1.64	2.05	1.46	1.20	1.43	2.28	2.10	1.00
1,2,3,4,7,8-Hexachlorodibenzofuran	5.66	5.33	6.30	6.00	5.73	6.32	5.69	5.16	6.06	4.58
1,2,3,6,7,8-Hexachlorodibenzofuran	2.03	2.04	1.87	3.20	2.64	3.88	2.96	2.79	3.13	2.82
2,3,4,6,7,8-Hexachlorodibenzofuran	0.23	0.27	0.25	0.15	0.30	0.31	0.30	0.18	0.24	0.25
1,2,3,7,8,9-Hexachlorodibenzofuran	1.33	1.50	2.20	2.83	1.88	1.85	1.01	0.99	2.08	2.75
1,2,3,4,7,8-Hexachlorodibenzo-p-dioxin	0.18	0.22	0.26	0.09	0.15	0.19	0.22	0.16	0.16	0.13
1,2,3,6,7,8-Hexachlorodibenzo-p-dioxin	0.31	0.69	0.73	0.56	0.79	0.34	0.63	0.61	0.38	0.75
1,2,3,7,8,9-Hexachlorodibenzo-p-dioxin	0.23	0.21	0.13	0.24	0.24	0.19	0.24	0.15	0.27	0.17
1,2,3,4,6,7,8-Heptachlorodibenzofuran	0.16	0.25	0.20	0.15	0.21	0.23	0.22	0.25	0.25	0.18
1,2,3,4,6,7,8-Heptachlorodibenzo-p-dioxin	0.10	0.11	0.08	0.10	0.08	0.06	0.07	0.10	0.05	0.07
1,2,3,4,7,8,9-Heptachlorodibenzofuran	0.02	0.04	0.04	0.05	0.03	0.04	0.03	0.03	0.02	0.03
Octachlorodibenzo-p-dioxin	<LOQ	<LOQ	<LOQ	<LOQ	<LOQ	<LOQ	<LOQ	<LOQ	<LOQ	<LOQ
Octachlorodibenzofuran	<LOQ	<LOQ	<LOQ	<LOQ	<LOQ	<LOQ	<LOQ	<LOQ	<LOQ	<LOQ
∑Congeners	16.28	18.91	20.61	20.56	18.86	**20.75**	19.79	17.46	18.58	20.20

The number given in bold is to show the highest sum between all.

**Table 12:** TEQ ng/kg for PCDD/F congeners in muscle samples.

	S1	S2	S3	S4	S5	S6	**S7**	S8	S9	S10
2,3,7,8-Tetrachlorodibenzofuran	1.24	1.52	0.86	1.24	0.98	1.61	1.80	0.77	0.83	0.91
2,3,7,8-Tetrachlorodibenzeno-p-dioxin	0.50	0.56	0.68	0.31	0.56	0.43	0.43	0.75	0.56	0.62
1,2,3,7,8-Pentachlorodibenzofuran	0.29	0.59	0.36	0.28	0.55	0.44	0.50	0.42	0.48	0.43
2,3,4,7,8-Pentachlorodibenzofuran	4.73	4.94	2.16	5.29	5.01	4.16	5.35	4.62	2.39	5.18
1,2,3,7,8-Pentachlorodibenzo-p-dioxin	1.24	1.30	1.18	1.30	1.37	1.12	1.06	1.12	0.93	1.24
1,2,3,4,7,8-Hexachlorodibenzofuran	1.10	0.95	1.27	0.86	1.53	1.03	0.94	0.91	1.31	0.76
1,2,3,6,7,8-Hexachlorodibenzofuran	1.09	1.04	1.09	1.76	0.90	1.02	0.91	1.70	1.65	0.86
2,3,4,6,7,8-Hexachlorodibenzofuran	0.13	0.09	0.07	0.11	0.14	0.15	0.08	0.09	0.14	0.12
1,2,3,7,8,9-Hexachlorodibenzofuran	0.71	0.81	0.57	0.60	0.93	1.23	1.07	0.63	0.47	1.06
1,2,3,4,7,8-Hexachlorodibenzo-p-dioxin	0.06	0.12	0.06	0.09	0.05	0.08	0.07	0.12	0.07	0.06
1,2,3,6,7,8-Hexachlorodibenzo-p-dioxin	0.11	0.13	0.14	0.12	0.09	0.17	0.12	0.17	0.17	0.14
1,2,3,7,8,9-Hexachlorodibenzo-p-dioxin	0.10	0.06	0.11	0.11	0.11	0.11	0.09	0.08	0.06	0.07
1,2,3,4,6,7,8-Heptachlorodibenzofuran	0.02	0.04	0.04	0.03	0.04	0.03	0.03	0.04	0.03	0.04
1,2,3,4,6,7,8-Heptachlorodibenzo-p-dioxin	0.01	0.01	0.01	0.01	0.01	0.01	0.01	0.01	0.01	0.01
1,2,3,4,7,8,9-Heptachlorodibenzofuran	0.07	0.14	0.12	0.07	0.13	0.10	0.10	0.07	0.14	0.16
Octachlorodibenzo-p-dioxin	<LOQ	<LOQ	<LOQ	<LOQ	<LOQ	<LOQ	<LOQ	<LOQ	<LOQ	<LOQ
Octachlorodibenzofuran	<LOQ	<LOQ	<LOQ	<LOQ	<LOQ	<LOQ	<LOQ	<LOQ	<LOQ	<LOQ
∑Congeners	11.41	12.30	<LOQ	12.19	12.39	11.69	**12.58**	11.50	9.23	11.67

The number given in bold is to show the highest sum between all.

**Table 13:** TEQ ng/kg for PCDD/F congeners in gizzard samples.

	S1	S2	**S3**	S4	S5	S6	S7	S8	S9	S10
2,3,7,8-Tetrachlorodibenzofuran	1.16	1.96	2.58	2.43	2.23	2.10	1.07	1.24	2.29	1.83
2,3,7,8-Tetrachlorodibenzeno-p-dioxin	0.68	0.93	0.56	0.37	0.37	0.81	0.62	0.37	0.31	0.93
1,2,3,7,8-Pentachlorodibenzofuran	0.52	0.43	0.66	0.63	0.61	0.71	0.89	0.50	0.43	0.76
2,3,4,7,8-Pentachlorodibenzofuran	4.73	4.32	9.52	8.40	9.96	4.55	10.62	4.60	4.77	5.80
1,2,3,7,8-Pentachlorodibenzo-p-dioxin	2.30	2.30	3.35	3.23	1.99	2.92	1.74	2.48	2.92	2.61
1,2,3,4,7,8-Hexachlorodibenzofuran	2.91	1.90	1.93	2.61	1.81	2.22	2.75	1.84	1.89	1.63
1,2,3,6,7,8-Hexachlorodibenzofuran	1.61	2.66	2.06	2.91	2.30	1.85	1.68	1.81	2.53	2.50
2,3,4,6,7,8-Hexachlorodibenzofuran	0.21	0.14	0.21	0.17	0.17	0.13	0.10	0.12	0.22	0.11
1,2,3,7,8,9-Hexachlorodibenzofuran	1.55	1.20	1.00	1.24	1.31	1.02	1.46	1.50	1.30	1.12
1,2,3,4,7,8-Hexachlorodibenzo-p-dioxin	0.18	0.15	0.11	0.06	0.15	0.16	0.14	0.07	0.08	0.16
1,2,3,6,7,8-Hexachlorodibenzo-p-dioxin	0.26	0.24	0.31	0.14	0.23	0.34	0.13	0.17	0.17	0.24
1,2,3,7,8,9-Hexachlorodibenzo-p-dioxin	0.09	0.11	0.18	0.17	0.09	0.06	0.14	0.08	0.11	0.14
1,2,3,4,6,7,8-Heptachlorodibenzofuran	0.01	0.01	0.02	0.02	0.01	0.01	0.01	0.01	0.01	0.01
1,2,3,4,6,7,8-Heptachlorodibenzo-p-dioxin	0.03	0.03	0.03	0.02	0.03	0.03	0.02	0.03	0.03	0.02
1,2,3,4,7,8,9-Heptachlorodibenzofuran	0.02	0.03	0.02	0.02	0.03	0.02	0.01	0.01	0.01	0.02
Octachlorodibenzo-p-dioxin	<LOQ	<LOQ	<LOQ	<LOQ	<LOQ	<LOQ	<LOQ	<LOQ	<LOQ	<LOQ
Octachlorodibenzofuran	<LOQ	<LOQ	<LOQ	< LOQ	<LOQ	<LOQ	<LOQ	<LOQ	<LOQ	<LOQ
∑Congeners	16.26	16.41	**22.54**	22.43	21.29	16.94	21.38	14.86	17.07	17.86

The number given in bold is to show the highest sum between all.

## Data Availability

The data that support the findings of this study are available within the article.
